# SGLT-2i—A Useful Tool for Real-Life Metabolic and Body Weight Control in Type 2 Diabetes Mellitus Patients

**DOI:** 10.3390/medicina61030548

**Published:** 2025-03-20

**Authors:** Mihaela-Simona Popoviciu, Teodor Salmen, Delia Reurean-Pintilei, Vlad Voiculescu, Anca Pantea Stoian

**Affiliations:** 1 Faculty of Medicine and Pharmacy, University of Oradea, 410073 Oradea, Romania; elapopoviciu@yahoo.com; 2 Doctoral School, “Carol Davila” University of Medicine and Pharmacy, 050474 Bucharest, Romania; 3 Department of Medical-Surgical and Complementary Sciences, Faculty of Medicine and Biological Sciences, “Ștefan cel Mare” University, 720229 Suceava, Romania; 4 Dermatology Department, “Carol Davila” University of Medicine and Pharmacy, 020021 Bucharest, Romania; vlad.voiculescu@umfcd.ro; 5 Diabetes, Nutrition and Metabolic Diseases Department, “Carol Davila” University of Medicine and Pharmacy, 050474 Bucharest, Romania; anca.stoian@umfcd.ro

**Keywords:** sodium-glucose loop transporter 2 inhibitors, type 2 diabetes mellitus, metabolic control, body weight reduction, obesity

## Abstract

*Background and Objectives:* Elevated blood sugar poses an increasingly significant challenge to healthcare systems worldwide. We aimed to assess the efficacy of the SGLT-2i class in achieving metabolic control in patients with T2DM within a real-world standard-of-care regimen. *Material and Methods:* A prospective analysis was conducted over 6 months including individuals receiving care in an outpatient department, with baseline assessments and follow-ups at 3 and 6 months. *Results:* A total of 280 patients were assessed, with a mean age of 63.69 ± 9.16, 53.9% of which were males, with a mean DM duration of 9.06 ± 5.64 years, and a DM duration varying from 6 months to 24 years. *Discussion:* Real-world evidence bridges the gap between guidelines and practice. It emphasizes the need to overcome clinical inertia in order to optimize patient outcomes and contributes to the body of evidence supporting the efficacy of fixed-dose SGLT-2i combinations in managing T2DM and associated comorbidities. *Conclusions:* We demonstrate the significant clinical and therapeutic impact of SGLT-2i in T2DM patients in a real-world setting. This class of medication not only positively influences glycemic and weight control but also reduces CV risk factors and visceral adiposity.

## 1. Introduction

Elevated blood sugar, whether in the range of prediabetes or overt type 2 diabetes mellitus (DM), poses an increasingly significant challenge to healthcare systems worldwide [1]. Various degrees of excess weight, often associated with the visceral disposition of adipose tissue, are present in more than 80% of individuals living with glucose dysregulations [2].

A tight metabolic control defined by low A1c hemoglobin (HbA1c) levels may effectively prevent the occurrence of severe complications in patients with type 2 DM (T2DM) or other glycemic dysregulation types; but, moreover, obtaining body weight reduction (BWR) remains an important pillar in such patients’ treatment regimens. Furthermore, significant BWR has been demonstrated to reverse DM natural evolution and enable cessation of DM medications, thus even achieving DM remission [2]. Obesity and, notably, excess abdominal disposition of adipose tissue are also key factors that must be addressed in the process of obtaining optimal metabolic control [3]. Despite its importance, obesity and central adiposity are often inadequately addressed in clinical practice. DM medications with the potential to facilitate weight loss are also under-prescribed, negatively impacting patients [4,5,6].

More than 800 million adults worldwide are living with DM, which represents a substantial increase from previous estimates, and what is more alarming is that it shows a positive trend [7]. This is largely attributed to rising obesity rates, poor dietary habits, and physical inactivity. T2DM is considered a component of metabolic syndrome, a cluster of conditions that increase the risk of heart disease, stroke, and other health complications. According to the International Diabetes Federation, one in four adults suffers from metabolic syndrome, with prevalence varying based on age, gender, ethnicity, demographic factors, and levels of physical activity [8,9].

Sodium-glucose cotransporter 2 inhibitors (SGLT-2i) typically reduce HbA1c levels by approximately 1% and improve fasting plasma glucose (FPG) levels. Their mechanisms, beyond the well-known ones involved in glucose-lowering—such as reducing oxidative stress, improving endothelial function, lowering blood pressure, modulating sodium and fluid balance, and influencing inflammation—may also be relevant in neurodegenerative disorders. Several preclinical studies suggest that these drugs may mitigate tau pathology and β-amyloid accumulation, which are key factors in Alzheimer’s disease. A well-documented outcome of SGLT-2i treatment is the effect on BWR, depending on the patient’s baseline body mass index (BMI) and impacting both the visceral and subcutaneous adipose tissue. This dual benefit, impacting glucose parameters and body weight, highlights the therapeutic potential of this class in addressing concurrently both hyperglycemia and obesity. Their optimal use demands adherence to clinical guidelines advocating for integrated management strategies. SGLT-2i have also reshaped the cardio-reno-metabolic approach in DM, heart failure (HF), and chronic kidney disease (CKD) treatment, demonstrating cardio- and renal-protective effects alongside glucose control by reducing mortality risk and improving cardiac and renal function. Cognitive decline shares common pathophysiological pathways with DM, cardiovascular (CV) disease, and dementia, and by impacting neuroinflammation, oxidative stress, and vascular dysfunction—key contributors to dementia progression—neuroprotection may also extend these metabolic benefits. There is evidence suggesting that SGLT-2i may have a role in delaying cognitive decline in patients with DM. Preclinical and observational studies indicate potential benefits in preserving cognitive function and slowing the progression of this disease, possibly through improved brain insulin signaling, better cerebral circulation, and modulation of neurodegenerative processes. These findings remain inconsistent; however, some studies show no significant cognitive benefit. Definitive conclusions would be drawn through large, long-term RCTs which are needed to determine whether the SGLT-2i class could be considered a viable therapeutic option for neuroprotection, independent of the benefits shown in the metabolic domain. Given their current widespread use in DM, nephrology, and CV care, investigating their role in dementia prevention could potentially provide a new benefit [10,11,12,13].

Current clinical practice recommendations in T2DM management focus on prioritizing the multifactorial approach to patient care. This includes control of metabolic, body weight, blood pressure and lipid parameters to minimize complication rates and sustain a high quality of life. Integrating these elements is of utmost importance for reducing cardiovascular (CV) events and all other DM-associated health risks. Data provided from real-world reports and medication usage patterns based on current international guidelines offer a more nuanced understanding of SGLT-2i effectiveness and safety profiles in practical, everyday settings, thereby confirming and adding to a more comprehensive evidence base for their use [14].

The primary objective of our study is to systemically assess the efficacy of the SGLT-2i class in achieving metabolic control, promoting BWR, and reducing abdominal obesity in patients with T2DM from a real-world setting that uses the standard-of-care treatment of T2DM.

## 2. Materials and Methods

A prospective analysis was conducted on consecutively admitted patients in the outpatient department of the Clinic Center for Diabetes, Nutrition, and Metabolic Disorders at Bihor County Emergency Hospital, Romania, between January and June 2021. The study adhered to the principles of the Declaration of Helsinki and received approval from the Institutional Ethics Committee of Bihor County Emergency Hospital (protocol number 25979, dated 21 November 2022). Of the 302 patients initially recruited, 10 discontinued their treatment, and 12 refused or were unable to attend baseline visits.

Our study included individuals aged 18 years and older who met the following criteria: HbA1c level ≥ 7.2%, BMI ≥ 25 kg/m^2^, an established diagnosis of T2DM for at least six months, and no prior exposure to SGLT-2i or other antidiabetic medications with weight loss potential. Participants were selected for the initiation of treatment with SGLT-2i, either as monotherapy or in fixed-dose combinations with metformin according to national guidelines which recommend removing metformin and adding iSGLT2, if patients are intolerant. All patients received standard-of-care treatment for concurrent comorbidities, alongside with general nutritional therapy performed in patients with T2DM and provided informed consent for participation in the study. Exclusion criteria encompassed individuals with other forms of diabetes, specifically type 1 diabetes mellitus or secondary diabetes, those on pre-existing insulin therapy, individuals with a normal BMI (<25 kg/m^2^), HbA1c levels below 7.2%, a history of malignancy, those with psychiatric or neurodegenerative disorders, pregnancy or lactation. Notably, during the study period, only empagliflozin and dapagliflozin were available for use as per Romanian regulatory approval. As per the study protocol, data extracted from patient records during initial, 3-month, and 6-month visits included demographic information (age and gender), clinical parameters (height, weight, BMI, and abdominal circumference), DM duration, and laboratory findings (HbA1c). We did not collect data about the renal function, such as creatinine. Data processing was performed using SPSS 20 software and Excel 2010 software. Mean values of the parameters, frequency ranges, standard deviations, statistical significance tests using the Student’s method (*t*-test), and χ^2^ were calculated. ANOVA was used to compare means, and the statistical significance level was 0.05, with a confidence interval: %CI 95. Correlations were performed on the entire dataset and Pearson’s coefficient was used. Sensitivity to change can be assessed in long-term observational studies. To measure sensitivity to change, we used the statistical calculation system “effect size” (ES).

## 3. Results

### 3.1. Group Characteristics

The 280 patients included in this study had a mean age of 63.69 ± 9.16, 53.9% were males, and had a mean DM duration of 9.06 ± 5.64 years, as shown in Table 1. Their DM duration varied from 6 months to 24 years, with a mean duration ranging from 7.28 to 9.92 years.

There were no significant differences between the two groups—those receiving SGLT-2i monotherapy and those on fixed-dose combinations of SGLT-2i + metformin—in terms of gender (*p* = 0.923), age (*p* = 0.329), or duration of DM (*p* = 0.079).

### 3.2. Metabolic Parameters

Table 2 represents the mean HbA1c values at the three visits: baseline, 3 months, and 6 months, alongside with ES and percentual reduction in HbA1c.

For all types of therapy, the ES on HbA1c was moderate at 3 months (0.50–0.80) and very good at 6 months (>0.80), as shown in Table 2. Also, percentual reduction in HbA1c at 3 months ranged between 13.41% (dapagliflozin 10 mg) and 17.78% (empagliflozin + metformin 12.5/1000 mg), while at the 6-month visit it ranged between 22.13% (empagliflozin 10 mg) and 26.37% (empagliflozin 25 mg), as shown in Table 2.

### 3.3. Body Weight and Abdominal Parameters

Table 3 represents the mean BMI values at the three visits: baseline, 3 months, and 6 months.

Table 4 presents the mean BWR values recorded at the follow-up visits, specifically at 3 months and 6 months. For all therapeutic variants, ES on BMI was non-significant at 3 months (ES < 0.20) and small at 6 months (0.20–0.49), as shown in Table 3. Percentual reduction in BMI at 3 months ranged between 1.53% (dapagliflozin 10 mg) and 2.19% (empagliflozin + metformin 12.5/1000 mg), while at 6 months it ranged between 3.23% (empagliflozin 25 mg) and 6.53% (empagliflozin + metformin 12.5/1000 mg), as shown in Table 3.

Table 5 represents the mean abdominal circumference difference between the follow-up visits, at 3 months and 6 months, respectively.

The ES and the percentual reduction in abdominal circumference are shown in Table 6.

When analyzing the impact of DM duration for the entire dataset, its increment negatively correlates with BMI (r = −0.22, *p* < 0.001), BWR (r = −0.273, *p* < 0.001) and abdominal circumference (r = −0.541, *p* < 0.001).

## 4. Discussion

This study evaluated the efficacy of SGLT-2i in a routine practice environment focusing on metabolic control, BWR, and abdominal obesity in patients with T2DM. The findings demonstrated improvements in glycemic control, as evidenced by HbA1c reductions across all treatment groups, regardless of the specific SGLT-2i or dose used. Furthermore, the results underlined the effects of SGLT-2i in facilitating weight loss and abdominal obesity reduction, particularly when used in fixed-dose combinations with metformin.

The majority of individuals included in our study were males (52.8–54.5%), with a mean age ranging from 61.29 to 65.09 years. According to global epidemiological studies, the prevalence of T2DM is reported to be slightly higher in men, especially in age categories over 60 years [15,16]. Moreover, this demographic group is also associated with multiple risk factors, such as sedentary behavior, obesity—particularly abdominal obesity—and age-related metabolic changes. The duration of T2DM ranged from 7.28 to 9.92 years, with no significant differences observed between groups. Typically, in patients with longer DM duration, insulin resistance (IR) [17,18,19,20,21,22] is more prevalent, negatively influencing responses to therapy and accelerating the development of chronic complications [23,24,25,26,27,28,29].

Regarding the SGLT-2i effect in recently diagnosed DM and in patients with longer disease duration, large clinical trials such as the DECLARE-TIMI 58 have demonstrated comparable outcomes across different disease durations. Notably, weight loss tends to be more significant in those with short-standing DM [30]. Future studies, including larger cohorts or a more detailed stratification, are needed across different populations, with variations in demographics and age groups to address this issue.

The mean baseline HbA1c in this study was >8%, reflecting insufficient glycemic control at the start of treatment. This result underscores possible clinical inertia, a well-known barrier in DM management, with often delayed therapy intensification even in the context of suboptimal glycemic control. A plethora of factors set the stage for clinical inertia, including hesitation to escalate therapy from both patients and clinicians, caution regarding potential side effects, and inconsistent follow-up with the same healthcare professional. These setbacks are prone to lead to prolonged periods of poor glycemic control, increasing the risk of developing DM complications. Early and aggressive interventions are needed to mitigate clinical inertia, particularly since effective agents like SGLT-2i have been demonstrated to improve long-term outcomes by reducing HbA1c and associated risks. Clinical trials such as EMPA-REG OUTCOME and CANVAS have shown the benefits of SGLT-2i in reducing HbA1c, including in patients with elevated baseline levels [31,32,33,34].

Furthermore, current guidelines recommend this drug class in T2DM patients regardless of their baseline HbA1c due to the additional benefits in cardio-reno-metabolic protection [14,35,36,37,38,39,40]. The reduction in HbA1c observed in our study cohort aligns with previously published data [41,42,43,44,45,46]. Among the patients treated with the fixed-dose combination of empagliflozin and metformin (12.5/1000 mg od), a more pronounced reduction in HbA1c was observed. This significant improvement may be attributed to individual variability in response and efficacy to SGLT-2i, a fact that could further explain the greater HbA1c reduction seen with this dual-drug formulation compared to monotherapy with either dapagliflozin, empagliflozin, or the fixed combination of dapagliflozin and metformin. Furthermore, the combination of SGLT-2i and metformin promotes more pronounced BWR due to their complementary and synergistic effects, including enhanced glucose elimination via urine mediated by SGLT-2i, and reduced hepatic glucose production through inhibition of gluconeogenesis and improved insulin sensitivity mediated by metformin [47,48].

The combined effect results in a greater calorie deficit and sustained BWR. Metformin enhances cellular glucose utilization and reduces fat storage, thus lowering IR, while SGLT-2i induce a compensatory decrease in plasma insulin levels. This reduction in circulating insulin minimizes its anabolic effects on adipose tissue, further contributing to BWR [49]. The combined effect reduces circulating insulin levels, which limits fat storage and promotes the use of fat as an energy source. Additionally, SGLT-2i enhance lipid oxidation and mobilize visceral fat by inducing mild ketosis, as they increase the body’s reliance on lipids for energy [50]. Meanwhile, metformin stimulates the activation of AMPK, promoting lipid burning and reducing hepatic lipogenesis. Together, these mechanisms result in less fat storage, contributing to a significant decrease in BMI and visceral fat. Another contributing factor is the impact on appetite and caloric intake. Metformin may reduce hunger by acting on the hypothalamus, potentially through modulation of appetite-regulating hormones, further amplifying BWR [51,52,53,54,55,56].

Data from large clinical trials involving T2DM patients have shown that both empagliflozin and dapagliflozin effectively reduce HbA1c levels. However, some studies suggest that empagliflozin may have a marginally greater effect on HbA1c reduction compared to dapagliflozin [57,58].

The reduction in HbA1c evidenced in our study was independent of the type of SGLT-2i, confirming the universal efficacy of this drug class in lowering HbA1c, regardless of the specific inhibitor or dose used. SGLT-2i reduce glycemia by increasing glucose excretion through urine in a non-insulin-mediated manner and independent of the degree of IR. No significant differences were observed between groups in this study concerning HbA1c reduction. These results could be contextualized by previous publications suggesting a potential biological limit to the extent to which a reduction in HbA1c can be achieved through this mechanism [59,60]. Most clinical studies (EMPA-REG, DECLARE-TIMI 58) have reported HbA1c reductions of approximately 0.5–1% at 6 months, consistent with the ones demonstrated in our study. The benefits and impact of HbA1c reduction in clinical practice are significant on population and patient levels. Even a modest reduction in HbA1c substantially impacts the risk of microvascular complications, such as renal and ocular impairment [61].

There are several factors which could influence our results: the reductions in the HbA1c value may be less variable between groups due to inclusion criteria (patients with baseline HbA1c > 7.2%) and the similarity in clinical characteristics of the patients (BMI, age, DM duration). Previously published data have suggested that SGLT-2i have greater efficacy in patients with higher baseline HbA1c levels [32]. Similar to our findings, the large EMPA-REG OUTCOME trial [32] showed a 0.54% HbA1c reduction at 6 months, with no major differences between subgroups. The CANVAS Program study [62] reported uniform HbA1c reductions, suggesting that the effect is similar across patients who share common clinical characteristics. In the setting of fixed-dose combinations with metformin, the cumulative effect may amplify HbA1c reduction due to complementary mechanisms, namely reduced hepatic glucose production and SGLT2-induced glucosuria as previously stated. The similar results observed across the groups from our study suggest that the selection of SGLT2-i and dose may not significantly impact efficacy, permitting flexibility in adapting treatment to patients’ needs.

The mean BMI in our study ranged between 33 and 35 kg/m^2^, with no significant differences between groups. SGLT-2i are recognized as having a significant impact on reducing BMI, and they are particularly effective in cases of excess weight [63]. Their effect on body weight supports the use of this drug class for patients with T2DM and obesity, as recommended by current guidelines. The elevated BMI values in our cohort further justify the use of SGLT-2i, with the results obtained in this study aligning with data reported in the literature. Diabetic myopathy, a complication of T2DM, can lead to exercise intolerance, reducing the likelihood of lowering BMI. Therefore, a rehabilitation program is recommended to prevent exercise intolerance and increase the chances of BMI reduction through physical activity in patients with T2DM [64].

The fixed-dose combination of empagliflozin 12.5 mg and metformin 1000 mg resulted in a greater reduction in BMI at both 3 and 6 months compared to other treatment groups, with the most pronounced decrease observed at the 6-month follow-up visit.

Possible explanations involve the caloric deficit of approximately 200–300 calories being lost daily through glycosuria [65]. Clinical studies like EMPA-REG OUTCOME confirm that adding empagliflozin to standard treatment regimens results in greater BWR than metformin alone or using other antidiabetic drugs. In some publications, it has been shown to impact incretin hormone levels, thus indirectly contributing to weight loss [66,67]. Associating an SGLT-2i with metformin produces complementary effects, maximizing BWR through dual mechanisms [68,69]. Recent meta-analyses [67,70] also indicate that combined treatments have a more pronounced effect on BMI reduction than monotherapy. Characteristics of patients treated with this fixed combination, such as higher initial BMI or pronounced visceral adiposity, may contribute to a more favorable response. Fixed-dose combinations offer additional advantages for patients with T2DM, who often present with obesity or overweight and require both glycemic and weight control interventions.

At the 3-month follow-up visits following SGLT-2i administration with or without metformin, differences in BMI reduction efficacy were observed. The smallest BWR was observed with dapagliflozin 10 mg (1.35 kg), compared to empagliflozin 10 mg (1.43 kg) and empagliflozin 25 mg, which showed a BMI reduction of 1.72 kg. The differences were more significant when comparing these results to those obtained through fixed-dose combinations, empagliflozin + metformin 12.5/1000 mg and dapagliflozin + metformin 5/1000 mg. The pharmacodynamic properties of dapagliflozin and empagliflozin suggest these differences. While both belong to the SGLT-2i class, subtle variations may exist. For example, empagliflozin has a higher affinity for SGLT2 receptors, resulting in greater glucose excretion and, consequently, a possible higher calorie loss compared to dapagliflozin [71,72]. Concerning the fixed-dose combination of dapagliflozin and metformin (5/1000 mg), patient outcomes have demonstrated greater improvements. Another point to be considered is that the patient profile receiving 10 mg dapagliflozin may include factors impeding weight loss, as advanced age is known to be linked with diminished metabolic responses. Furthermore, prolonged DM duration and older age are often associated with different degrees of renal dysfunction, possibly decreasing the responsiveness to SGLT-2i. The fixed-dose combinations evaluated in our study were significantly more effective in reducing BMI. The results support current guidelines, which recommend the use of fixed-dose combinations in patients with severe obesity and poorly controlled DM to maximize therapeutic effects [14]. Similar aspects were observed at 6-month post-treatment follow-up visits.

Previous publications demonstrated a positive impact of SGLT-2i in reducing ectopic adiposity and improving adipose tissue function [73]. Another important aspect reflected in our results is the notable change in abdominal circumference, as evaluating adipose tissue distribution offers a more accurate reflection of IR than BMI [74,75]. In females, the greatest reduction was observed with fixed-dose combinations, which may indicate a cumulative impact of combined therapy on central adiposity [76]. In males, treatment with dapagliflozin or the fixed-dose combination of empagliflozin and metformin significantly reduced abdominal circumference over 6 months. These findings are consistent with the existing literature [77]. We found that, regardless of the type of therapy, the average reduction in abdominal circumference was greater in males than in females (*p* < 0.001). This more pronounced effect in males than females may be due to biological differences in the distribution of central adipose tissue and the response to the treatments that impact glycemic levels while also managing weight. We performed a comparative analysis between cohorts, which revealed that the fixed-dose combinations of empagliflozin + metformin (12.5/1000 mg) and dapagliflozin + metformin (5/1000 mg) resulted in significantly greater reductions in abdominal circumference at 6 months. Specifically, reductions were observed in both females (4.16 cm versus 3.94 cm) and males (11.83 cm versus 10.75 cm). Among males receiving SGLT-2i monotherapy, empagliflozin 25 mg was the most effective, achieving a reduction of 10.26 cm at 6 months. These differences in the obtained outcomes between genders underline the necessity for individualized treatment approaches and are of clinical importance, as abdominal adiposity serves as a clinical marker of CV risk, and its reduction contributes to improved clinical prognosis of patients with T2DM. Reducing CV risk in patients with T2DM is considered a primary objective in therapeutic management. SGLT-2i have demonstrated efficacy across all stages of T2DM through their insulin-independent mechanisms [30,32,38]. Previous research has demonstrated the beneficial effects of SGLT-2i on abdominal obesity in patients with T2DM [38]. These benefits are evident both in monotherapy and when combined with other oral antidiabetic agents or insulin, contributing to the reduction in abdominal adiposity [78,79,80,81,82]. When analyzing correlations, we observed that the patients with a shorter duration of DM experienced greater benefits in waist circumference reduction. Similarly, the DM duration showed a significant negative correlation with the reduction in abdominal circumference, suggesting a better therapeutic response in patients with recently diagnosed DM. These correlations suggest that early interventions with SGLT-2i facilitate more substantial benefits in reducing abdominal adiposity.

The pleiotropic effects of SGLT-2i play a significant role in CV, renal, and metabolic health, with their mechanisms extending beyond glucose regulation and including lipid oxidation, improved insulin sensitivity, and modulated inflammation, all of which may contribute to their broader therapeutic benefits, including CV and renal protection [83]. Incorporating SGLT-2 inhibition into a patient’s treatment regimen shifts the body’s primary energy source from glucose to lipid oxidation, leading to improved myocardial efficiency and protection against ischemic injury in cardiac myocytes. Additionally, this shift helps reduce oxidative stress and inflammation, two key contributors to cardiovascular and metabolic diseases [84]. By lowering blood glucose levels through renal glucose excretion, SGLT-2i also promote modest BWR, primarily from visceral adipose tissue, in turn reducing insulin resistance and glucotoxicity and ultimately benefiting vascular function. Furthermore, studies suggest that SGLT-2i influence the biology of epicardial preadipocytes and may suppress the expression and secretion of IL-6 in human epicardial tissue, highlighting their potential anti-inflammatory effects in the CV system [85].

SGLT-2i were initially prescribed for their glucose-lowering and BWR effects in T2DM, objectives attained primarily through urinary glucose excretion, with a corresponding energy deficit of approximately 300–360 kcal/day. Yet, there was consistently 50–75% less weight loss than predicted thus suggesting the implication of compensatory mechanisms, including potential effects on appetite regulation [86]. Evidence from different publications indicates that SGLT-2i therapy may lead to increased energy intake, with empagliflozin associated with a 13% rise in caloric consumption and canagliflozin linked to an intake increase of ~100 kcal/day per kilogram of weight loss, data that have been also supported by non-human experimental studies [87,88,89,90]. Energy balance is regulated through gut peptides such as PYY and GLP-1, which promote satiety, and acylated ghrelin conversely stimulating hunger. The hypothesis was that the weight loss induced by these drugs could impact the patterns of secretion of these hormones, driving compensatory eating [91]. However, robust human data remains limited, and the small, uncontrolled studies have concluded mixed results. The SEESAW trial aimed to clarify this aspect by evaluating changes in postprandial appetite-regulatory peptides over 24 weeks of SGLT-2i therapy, dietary energy restriction, or both. No significant differences were found in PYY, GLP-1, or ghrelin levels between groups [92]. Thus, up to date, the direct effects of SGLT-2i on appetite regulation remain inconclusive. The hedonic appetite control as well as the compensatory eating behaviors and long-term energy balance adaptations should be addressed by future research to understand the underlying mechanisms for the attenuated weight loss observed with these therapies.

### Study Limitations and Future Perspectives

Limitations of the present study are absence of a long-term analysis (12–24 months), a relatively small number of patients that prevents us from a more thorough analysis between groups and a lack of analysis of the patients’ comorbidities.

Future perspectives include larger real-life long-term analyses and studies in order to confirm the beneficial results of different SGLT-2i therapies in different doses, as well as fixed combinations in different doses, alongside additional biomarker analysis for a better understanding of the mechanisms of action in the long term. Also, numerous RCTs, metanalysis, and real-world reports are positioning this medication class as fundamental in reducing the risk of HF and CKD progression regardless of the presence of DM. In patients with or without DM and stage 3 to 5 CKD, HF is the most frequent CV event [93]. SGLT-2i are now a class of medication shared between medical specialties due to their protective effects apart from glucose lowering. Prescription protocols integrate them in the treatment for both preserved and reduced ejection fraction HF as well as in reducing the urinary elimination of albumin and slowing the decline in eGFR slopes. It is interesting to notice that CKD progression is impacted independently of the initial eGFR; however, the effect is larger if the treatment is initiated at higher eGFR values [94,95].

## 5. Conclusions

This study demonstrates the significant clinical and therapeutic impact of SGLT-2i, particularly fixed-dose combinations, in T2DM patients, often associated with excess weight in a real-world setting. This class of medication not only positively influences glycemic and weight control, but also reduces CV risk factors and visceral adiposity, offering a clear and sustained therapeutic advantage. Given the multifaceted interactions between obesity, DM, and CV risk factors, early initiation of SGLT-2i is essential. Real-world evidence bridges the gap between guidelines and practice, reiterates the need to overcome clinical inertia to optimize patient outcomes, and contributes to evidence supporting the efficacy of fixed-dose SGLT-2i combinations in managing T2DM and associated comorbidities.

## Figures and Tables

**Table 1:** Group characteristics stratification after gender, age, and DM duration.

Parameter	Value	*p*-Value
Gender (Female/Male)		
Dapagliflozin 10 mg	16 (47.1%)/18 (52.9%)	0.965
Empagliflozin 10 mg	40 (45.5%)/48 (54.5%)	
Empagliflozin 25 mg	25 (47.2%)/28 (52.8%)	
Empagliflozin 12.5 mg/Metformin 1000 mg	25 (45.5%)/30 (54.5%)	
Dapagliflozin 5 mg/Metformin 1000 mg	23 (46.0%)/27 (54.0%)	
Monotherapy with SGLT-2i	81 (46.3%)/94 (53.7%)	0.923
Fixed dose combinations with SGLT-2i + Metformin	48 (45.7%)/57 (54.3%)	
Age (41–80 years)		
Dapagliflozin 10 mg	61.29 ± 6.73 years	0.392
Empagliflozin 10 mg	62.94 ± 9.22 years	
Empagliflozin 25 mg	65.09 ± 8.22 years	
Empagliflozin + Metformin 12.5/1000 mg	64.45 ± 10.78 years	
Dapagliflozin + Metformin 5/1000 mg	64.30 ± 9.38 years	
Monotherapy with SGLT-2i	63.27 ± 8.55 years	0.329
Fixed dose combinations with SGLT-2i + Metformin	64.38 ± 10.09 years	
DM duration (6 months–24 years)		
Dapagliflozin 10 mg	9.65 ± 5.21 years	0.177
Empagliflozin 10 mg	9.23 ± 6.06 years	
Empagliflozin 25 mg	9.92 ± 5.75 years	
Empagliflozin + Metformin 12.5/1000 mg	7.28 ± 4.54 years	
Dapagliflozin + Metformin 5/1000 mg	8.42 ± 5.90 years	
Monotherapy with SGLT-2i	9.52 ± 5.78 years	0.079
Fixed dose combinations with SGLT-2i + Metformin	8.30 ± 5.32 years	

SGLT-2i—Sodium-glucose cotransporter 2 inhibitors.

**Table 2:** The mean (%) variation, ES, and percentual reduction in HbA1c between the visits.

	Baseline	3-Month Visit	6-Month Visit	*p*-Value	3-Month ES	6-Month ES	3-Month Reduction (%)	6-Month Reduction (%)
Dapagliflozin 10 mg	8.65 ± 1.68	7.49 ± 1.28	6.68 ± 1.01	<0.001	0.69	1.17	−13.41	−22.77
Empagliflozin 10 mg	8.81 ± 1.94	7.54 ± 1.31	6.86 ± 1.14	<0.001	0.65	1.01	−14.42	−22.13
Empagliflozin 25 mg	9.14 ± 2.47	7.53 ± 1.10	6.73 ± 0.71	<0.001	0.65	0.98	−17.61	−26.37
Empagliflozin + Metformin 12.5/1000 mg	8.72 ± 2.02	7.17 ± 0.88	6.61 ± 0.85	<0.001	0.77	1.04	−17.78	−24.20
Dapagliflozin + Metformin 5/1000 mg	8.89 ± 1.86	7.56 ± 1.52	6.81 ± 1.19	<0.001	0.72	1.12	−14.96	−23.40
*p*-value	0.665	0.833	0.636					
Monotherapy with SGLT-2i	8.88 ± 2.07	7.53 ± 1.24	6.79 ± 1.00	<0.001	0.65	1.01	−15.20	−23.54
Fixed dose combinations with SGLT-2i + Metformin	8.80 ± 1.94	7.36 ± 1.23	6.70 ± 1.03	<0.001	0.74	1.08	−16.36	−23.86
*p*-value	0.763	0.263	0.501					

SGLT-2i—Sodium-glucose cotransporter 2 inhibitors; ES—effect size.

**Table 3:** The mean (kg/m^2^) reduction, ES, and percentual variation of BMI between the visits.

	Baseline	3-Month Visit	6-Month Visit	*p*-Value	3-Month ES	6-Month ES	3-Month Reduction (%)	6-Month Reduction (%)
Dapagliflozin 10 mg	34.03 ± 4.56	33.51 ± 4.50	32.88 ± 4.52	0.300	0.11	0.25	−1.53	−3.38
Empagliflozin 10 mg	33.74 ± 4.68	33.24 ± 4.62	32.58 ± 4.55	0.097	0.11	0.25	−1.48	−3.44
Empagliflozin 25 mg	34.65 ± 7.02	34.14 ± 6.86	33.53 ± 6.67	0.127	0.07	0.16	−1.47	−3.23
Empagliflozin + Metformin 12.5/1000 mg	33.83 ± 6.22	33.09 ± 6.31	31.62 ± 6.41	0.069	0.12	0.36	−2.19	−6.53
Dapagliflozin + Metformin 5/1000 mg	34.08 ± 5.20	33.36 ± 5.02	32.50 ± 5.00	0.125	0.14	0.30	−2.11	−4.64
*p*-value	0.348	0.335	0.306					
Monotherapy with SGLT-2i	34.07 ± 5.51	33.57 ± 5.41	32.93 ± 5.29	0.049	0.09	0.21	−1.47	−3.35
Fixed dose combinations with SGLT-2i + Metformin	33.95 ± 5.73	33.22 ± 5.71	32.20 ± 5.76	0.026	0.13	0.31	−2.15	−5.15
*p*-value	0.862	0.608	0.281					

SGLT-2i—Sodium-glucose cotransporter 2 inhibitors; ES—effect size.

**Table 4:** The mean BWR (kg) between the visits.

	3-Month Visit	6-Month Visit
Dapagliflozin 10 mg	1.35 ± 0.88	3.00 ± 1.44
Empagliflozin 10 mg	1.43 ± 0.91	3.30 ± 1.75
Empagliflozin 25 mg	1.72 ± 1.18	4.30 ± 2.84
Empagliflozin + Metformin 12.5/1000 mg	2.05 ± 1.13	5.29 ± 2.66
Dapagliflozin + Metformin 5/1000 mg	1.96 ± 1.43	4.38 ± 2.81
*p*-value	0.003	<0.001
Monotherapy with SGLT-2i	1.50 ± 0.99	3.54 ± 2.14
Fixed dose combinations with SGLT-2i + Metformin	2.01 ± 1.27	4.66 ± 2.75
*p*-value	<0.001	<0.001

SGLT-2i—Sodium-glucose cotransporter 2 inhibitors.

**Table 5:** The mean abdominal circumference (cm) reduction between the visits.

	Females		Males		* *p*-Value
	3-Month Visit	6-Month Visit	3-Month Visit	6-Month Visit	
Dapagliflozin 10 mg	1.18 ± 1.17	3.46 ± 2.62	5.59 ± 1.69	9.40 ± 2.02	<0.001
Empagliflozin 10 mg	1.43 ± 1.81	3.24 ± 2.52	5.63 ± 1.59	9.70 ± 2.39	<0.001
Empagliflozin 25 mg	1.95 ± 2.21	4.12 ± 2.62	5.81 ± 1.29	10.26 ± 2.30	<0.001
Empagliflozin + Metformin 12.5/1000 mg	2.13 ± 1.57	4.16 ± 1.60	6.56 ± 0.78	11.83 ± 2.20	<0.001
Dapagliflozin + Metformin 5/1000 mg	1.98 ± 1.99	3.94 ± 2.41	6.20 ± 0.87	10.75 ± 1.94	<0.001
*p*-value	0.314	0.467	0.022	<0.001	
Monotherapy with SGLT-2i	1.54 ± 1.75	3.56 ± 1.69	5.68 ± 1.29	9.81 ± 2.39	<0.001
Fixed dose combinations with SGLT-2i + Metformin	2.06 ± 1.81	4.05 ± 2.44	6.39 ± 1.59	11.32 ± 2.30	<0.001
*p*-value	0.314	0.467	<0.001	<0.001	

SGLT-2i—Sodium-glucose cotransporter 2 inhibitors. * *p*-value at 3 and 6 months.

**Table 6:** The ES and percentual reduction in the abdominal circumference (cm) between the visits.

	ES				Percentual Reduction			
	Females		Males		Females		Males	
	3-Month Visit	6-Month Visit	3-Month Visit	6-Month Visit	3-Month Visit	6-Month Visit	3-Month Visit	6-Month Visit
Dapagliflozin 10 mg	0.08	0.20	0.23	0.41	−1.48	−3.57	−3.54	−6.47
Empagliflozin 10 mg	0.12	0.28	0.61	1.57	−1.49	−3.38	−5.19	−13.42
Empagliflozin 25 mg	0.19	0.40	0.28	0.82	−1.94	−4.10	−4.86	−14.06
Empagliflozin + Metformin 12.5/1000 mg	0.10	0.22	0.58	0.56	−2.22	−4.92	−5.54	−5.36
Dapagliflozin + Metformin 5/1000 mg	0.13	0.27	0.87	0.66	−2.20	−4.53	−5.61	−4.26
Monotherapy with SGLT-2i	0.11	0.24	0.14	0.32	−1.64	−3.64	−1.72	−3.91
Fixed dose combinations with SGLT-2i + Metformin	0.12	0.36	0.30	0.58	−1.77	−5.05	−2.77	−5.43

SGLT-2i—Sodium-glucose cotransporter 2 inhibitors. ES—effect size.

## Data Availability

Dat available on request from the authors.

## Abbreviations

The following abbreviations are used in this manuscript:

BMI	Body Mass Index
BWR	Body Weight Reduction
CV	Cardiovascular
DM	Diabetes Mellitus
FPG	Fasting Plasma Glucose
HbA1c	A1c Hemoglobin
SGLT-2i	Sodium-glucose Cotransporter 2 inhibitors
T2DM	Type 2 Diabetes Mellitus
